# A Five-year Study of Spinal Disorders Among Patients Presenting to the National Trauma Center of Nepal: An Observational Study

**DOI:** 10.31729/jnma.8809

**Published:** 2024-11-30

**Authors:** Badri Rijal, Min Chandra Adhikari, Suzit Bhusal, Reshika Shrestha, Ashlesha Chaudhary, Dipendra Pandey, Mandish Prasad Phuyal, Akanshya Prasai, Aashutosh Chaudhar

**Affiliations:** 1 Department of Orthopedics, National Trauma Center, Mahankal, Kathmandu, Nepal; 2 Department of Medicine, National Trauma Center, Mahankal, Kathmandu, Nepal; 3 Research and Development Unit, National Trauma Center, Mahankal, Kathmandu, Nepal; 4 Maharajgunj Medical Campus, Maharajgunj, Kathmandu, Nepal; 5 Kathmandu Medical College and Teaching Hospital, Sinamangal, Kathmandu, Nepal; 6 Kathmandu University School of Medical Sciences, Dhulikhel, Kavre, Nepal

**Keywords:** *spinal cord injuries*, *spinal diseases*, *spine*

## Abstract

**Introduction:**

Spinal cord injuries result in severe neurological impairments and disabilities. With an estimated 15.4 million cases globally in 2021, spinal cord injuries are more common in low- and middle-income countries, yet research in these areas is limited. This study aimed to find the pattern of spinal injuries and outcomes associated with spine injuries over a five-year duration at a tertiary trauma care center.

**Methods:**

This observational cross-section study was conducted at the National Trauma Center, Kathmandu from 2075 to 2080 B.S. with ethical clearance from the Nepal Health Research Council (Reference number: 968). Total-population sampling was used. A structured proforma was employed as the primary data collection tool. Data was analyzed using SPSS.

**Results:**

Of the 20843 patients, 2070 (9.93%) had spinal injuries. The median age was 43 (IQR 32-56) years, with 1391 (67.20%) male patients. The median hospital stay was 12 (IQR 7-20) days. Falls accounted for 1221 (58.99%) cases, and road traffic accidents for 195 (9.42%). Spinal fractures were present in 1076 (51.98%) patients with 456 (42.38%) in lumbar vertebra. There were 225 (10.87%) cases of subluxation with 214 (95.11%) in cervical level.

**Conclusions:**

The study provides insights into the patterns and outcomes of spinal injuries over five years of time. The mortality rate and cases of patients leaving against medical advice highlight areas for improvement in patient care and follow-up.

## INTRODUCTION

Spinal cord injuries (SCIs) are intricate medical conditions brought on by assault to the spinal cord, often caused by trauma, such as falls and motor vehicle accidents, as well as non traumatic causes, such as malignancy and degeneration.^1^ This condition often results in severe neurological impairments and disabilities, causing loss of sensory and motor functions (paraplegia or tetraplegia), frequent infections in the bladder and kidneys, bowel problems, and cardiac and respiratory dysfunctions.^2^

An estimated 15.4 million persons worldwide were estimated to have SCI in 2021.^3^ Compared to the high-income countries the incidence of traumatic spinal cord injuries was hgher in low- and middle-income countries.^4^ Despite the high incidence of spinal cord injuries, very few studies have been done to highlight the burden of spinal cord injuries in low- and middle-income countries.

This study aimed to find the pattern of spinal injuries and outcomes associated with spine injuries through a meticulous five-year analysis of spinal injuries at a tertiary trauma care center.

## METHODS

This observational cross-section study was done at the National Trauma Center, Kathmandu. This is the only tertiary care trauma referral center of Nepal. The study was done after getting ethical clearance from the Nepal Health Research Council (NHRC) (Reference number: 968). All patients admitted to the National Trauma Center within the study timeframe were included. Patients brought dead were excluded from the study. Data was collected from the electronic record section of the National Trauma Center. This study used total-population sampling, where the sample size equaled the entire population since all relevant patient records within the specified timeframe were included. Adopting a census approach, data from all individuals admitted to the National Trauma Center over the past five years from 2075 to 2080 were analyzed. Duplicate records and those with missing data were excluded from the analysis. Thus, a total of 20843 cases data were taken in the study.

A structured proforma was employed as the primary data collection tool. Data collection involved a careful review and extraction of medical records from the National Trauma Center's database, adhering to ethical guidelines to maintain patient confidentiality. The collected data were verified for completeness and organized for subsequent analysis. Statistical analysis was carried out using SPSS, employing descriptive statistics such as mean, median, frequency, standard deviation (SD), interquartile range (IQR) and percentage.

## RESULTS

The total number of patients with spinal injuries was 2070 (9.93%). The median age of the patient admitted with spinal injuries was 43 (IQR 32-56) years. There were 1391 (67.20%) male and 679 (32.80%) females. The median of hospital stay was 12 (IQR 7-20) days. Among these patients, fall injury accounted for 1221 (58.99%) cases. There were 195 (9.42%) cases of road traffic accidents and 40 (1.93%) cases of blunt trauma. The spinal fracture accounted to 1076 (51.98%) cases with 225 (10.87%) cases of subluxation and 177 (8.55%) cases of disc prolapse (Table 1).

**Table 1:** Types of spinal injuries admitted in National Trauma Center (n=2070).

Spinal Injuries	n (%)
Fracture	1076 (51.98)
Subluxation	225 (10.87)
Disc prolapse	177 (8.55)
Translation injury	93 (4.49)
Disc degeneration	91 (4.40)
Spondylolisthesis	67 (3.24)
Infection	61 (2.95)
Dislocation	61 (2.95)
Myelopathy	46 (2.22)
Stenosis	40 (1.93)
Herniation	11 (0.53)
Contusion	10 (0.48)
Spondylosis	7 (0.34)
Radiculopathy	6 (0.29)
Central cord compression	5 (0.29)
Central cord syndrome	4 (0.19)
Abscess	1 (0.05)
miscellaneous	89 (4.3)

Among the admitted patients, 1756 (84.83%) were discharged after recovery, 75 (3.62%) left against medical advice (LAMA), and 42 (2.03%) resulted in mortality (Table 2).

**Table 2:** Outcomes of patients with spinal injuries admitted (n=2070).

Outcomes	n (%)
Recovered	1756 (84.83)
Not improved	110 (5.31)
LAMA	75 (3.62)
Referred	25 (1.21)
DOPR	61 (2.95)
Death	42 (2.03)
Absconded	1 (0.05)

LAMA=Left against medical advice, DOPR=Discharged on patient's request

Among spinal injuries, the spinal fracture in lumbar vertebra was found to be 456 (42.38%), 360 (33.46%) in thoracic vertebra, and 248 (23.05%) in cervical vertebra. The vertebral subluxation in cervical vertebra accounted to 214 (95.11%) and the prolapse in lumbar vertebra accounted to 84 (47.46) (Table 3).

**Table 3:** Level of spinal injury based on type of injury (n=2070).

Spinal Level	Fracture n(%)	Subluxation n(%)	Prolapse n(%)
Cervical vertebra	248 (23.05)	214(95.11)	23 (12.99)
Cervicothoracic	3 (0.28)	6(2.67)	-
Thoracolumbar	5 (0.46)	-	-
Lumbar vertebra	456 (42.38)	-	84 (47.46)
Lumbosacral level	2 (0.19)	1 (0.44)	67 (37.85)
Sacral vertebra	2 (0.19)	-	-
Thoracic vertebra	360 (33.46)	4(1.78)	3 (1.69)
Total	1076	225	177

## DISCUSSION

In this study, we included a total of 2070 patients presenting to the National Trauma Center over five years. Out of the total admitted patients, 58.99% patients suffered from fall injury which was the most common mechanism of injury in our study. A similar finding was observed in a systematic review conducted in Nepal where fall injury was found to be the commonest cause of SCI amounting to 60% cause of total injuries.^5^ Also, another study in eastern Nepal showed fall injury as the most common mechanism of injury (37.86%).^6^ However, our finding is inconsistent with the data of the United States which reported 30% of spinal cord injuries whose mechanism was fall injury.^1^ This discrepancy may be due to the mountainous terrain of the country, the tendency of Nepalese people to climb trees to gather fodder for cattle. The percentage of spine injuries due to Road Traffic Accident (RTA) was found to be 9.42%. The systematic review done by Parajuli et. al found RTA to be the second most common etiology of SCI (composite mean as 17%).^5^ In a study conducted across seven Latin American nations, 40.81% of patients sustained spinal cord injury as a result of RTAs.^7^ RTA is the leading cause of spinal injury in the West, mostly affecting younger age groups. However, falls from trees or heights impact all age groups participating in livelihood more equitably.^8^

The mean length of hospital stay in patients with spinal cord injury was 13.5±9.7 months in a study done in Korea.^9^ In our study the median duration of hospital stay was 12 (IQR= 7-20) days. However, in a five-year longitudinal review of patterns of traumatic spinal cord injury done in India, the mean length of stay in hospital after spinal injury was 8.9 days.^10^ Here, there is a significant difference in hospital stay in our study and the study done in Korea in patients with similar illnesses. According to Jang et al., the longer duration of hospital stay in the study could be the problems with patient mobility, which are made worse by Korean home design, and the absence of rehabilitation and welfare services that assist released patients with SCI in returning to their regular life.^9^ Since our study was done in a tertiary care center where rehabilitation and physiotherapy facilities are available, it could have resulted in a shorter duration of hospital stay. Further, financial constraints also could have obliged the families to discontinue hospital treatment early.

In a study done by Khan SU et al. in Pakistan, thoracolumbar spine injury was the most common level of injury seen in 49.6% of patients followed by cervical spine injury which was seen in 29.9% of patients.^11^ In contrast to this our study showed spine injury in the lumbar region as the most common level of injury present in of total population followed by cervical level. A study done in Saudi Arabia showed findings similar to our study where the most common spine disorders affected the lumbar spine (53.1%) followed by a cervical spine (27.1%).^12^ The variation in the prevalence of spinal injury levels in these studies could be influenced by differences in demographics, trauma mechanisms, and healthcare systems. In our study, among spinal injuries, the spinal fracture in the lumbar vertebra was found to be 42.38% and 33.46% in the thoracic vertebra. In a study done in Eastern Nepal, the thoracic and lumbar SCIs were associated with spinal fractures in 100% and 85% of cases respectively.^6^ In a retrospective study done in western Nepal, the age ranged from 19 to 78 years with the mean age being 47.5 years. This is similar to our study where the median age of the sample population was 43 (IQR 32-56) years.^13^ In our study, there were 67.20% male and 32.80% female. The higher proportion of males is consistent with other studies done in various countries.^6,8,11,14^ Our study shows a mortality rate of 2.03% due to spinal cord injury. A study done in a low-resource setting had a 15.2% in-hospital mortality rate for SCI.^15^ However, this study included only the traumatic causes of spinal cord injury while our study included all cases of spinal cord injury regardless of etiology.

Evidence-based health care planning and resource allocation are based on precise and current estimates of the incidence and prevalence of illnesses. Because no effective curative treatment for individuals with SCI has been identified, prevention is essential, and the first step in achieving this aim would be to look at epidemiological trends.^16^

Being an observational cross-section study, it provides information about the characteristics of spinal injuries but does not establish causal relationships. Certain key demographic information, including patients' heights, weights, smoking histories, alcohol intake, and occupations, was missing from the dataset. There was incomplete data on coexisting conditions, such as other health disorders or comorbidities in the records. Multifactorial association on patients outcomes was beyond the scope of this study.

## CONCLUSIONS

This five-year analysis provides an overview of the patterns of spinal injuries in Nepal, highlighting falls as the predominant cause, probably due to unique occupational and environmental factors. The study reveals that lumbar and cervical injuries are most common, with a higher incidence in males. The level and pattern of spine injuries depicted by this study add on to the literature to tailor treatment approaches.
